# Decompression effects on bone healing in rat mandible osteomyelitis

**DOI:** 10.1038/s41598-021-91104-7

**Published:** 2021-06-03

**Authors:** Buyanbileg Sodnom-Ish, Mi Young Eo, Ji Hye Oh, Mi Hyun Seo, Hoon Joo Yang, Jong Ho Lee, Soung Min Kim

**Affiliations:** Department of Oral and Maxillofacial Surgery, Dental Research Institute, School of Dentistry, Seoul National University, 101 Daehak-ro, Jongno-gu, Seoul, 03080 Korea

**Keywords:** Diseases, Medical research

## Abstract

Osteomyelitis (OM) of the jaw is usually caused by a chronic odontogenic infection. Decompression is the release the intraluminal pressure in the cystic cavity allowing gradual bone growth from the periphery. The aim of this study was to analyze the effectiveness of decompression in an OM jaw model. A 4-mm-diameter defect was made on mandibles of fourteen Sprague–Dawley rats and inoculated with *S. aureus* (20 μl of 1 × 10^7^ CFU/ml) injection. Two weeks later, four groups were made as non-treatment (C1), only curettage (C2), curettage and decompression (E1), and curettage and decompression with normal saline irrigation (E2). After four weeks, each group was analyzed. Most micro-CT parameters, including bone mineral density [0.87 (± 0.08) g/cm^3^] with bone volume [0.73 (± 0.08) mm^3^] was higher in E2 group than that of C1 group (*p* = 0.04, *p* = 0.05, respectively). E2 group in histology showed the highest number of osteocytes than those of control groups, 91.00 (± 9.90) (*p* = 0.002). OPN were expressed strongly in the E1 (“5”: 76–100%) that those of other groups. Decompression drains induced advanced bone healing compared to that of curettage alone. Therefore, it could be recommended to use decompressive drain for enhancing the jaw OM management.

## Introduction

Osteomyelitis (OM) of the jaw is an inflammatory process that starts in the medullary space of the bone and progresses to cortical bone, the Haversian system, periosteum, and overlying soft tissue. This is usually caused by micro-organism infection into the bone tissues due to a trauma or odontogenic infection^1^. The gram-positive pathogen *Staphylococcus aureus* (*S. aureus*) is the most common OM causative agent in both children and adults^2^. The literature on OM of the jaw is extensive, and vast terminologies and classifications are used to describe this disease. Chronic non-bacterial OM is a rare non-infectious autoinflammatory bone disorder of unknown etiology, that occurs in all ages with 7 to 12 years peak onset with female predominance^3^.

The treatment of jaw OM in the literature is classified as surgical and non-surgical, while the aim differs depending whether or not bacterial infection is apparent^4^. The universally acknowledged and effectual treatment is a combination of antibiotic therapy and surgery consisting of sequestrectomy, saucerization, decortication, and closed-wound suction irrigation^5^. The surgical therapy approach has three main goals, which includes decompression and drainage of intramedullary pressure and subperiosteal abscesses caused by the osteomyelitic effect, surgical treatment of infected tissue and removal of infectious foci, and grafting healthy bone tissue into the infected area^6^.

By definition, decompression is a technique that creates a small opening in the cystic wall using surgical drains for drainage that releases intraluminal pressure that causes cystic reduction and permits gradual bone growth from the periphery^7^. Beside cystic reduction purpose, decompression is also applied to the management of chronic suppurative osteomyelitis of the jaw and bisphosphonate-related osteonecrosis of mandible^8,9^. The postoperative exudate obtained from the wound using decompression showed increase in macrophage activation, angiogenesis and osteogenesis related proteins, downregulation of interleukin (IL) 10 and upregulation of tumor necrosis factor-α (TNF-α), IL-1, -6, -8, and -28 through quantitative analysis using immunoprecipitation high-performance liquid chromatography with minimal error range less than 5%^9^. The mechanism of decompression in jaw OM postoperative management is pressure releasing, removing postoperative inflammatory product, and allowing gradual bone growth. In the progression of OM, most of bacterial invasion induces cascade of inflammatory host responses that lead to hyperemia, increased capillary permeability, and local inflammation of granulocytes. During this host response, proteolytic enzymes are released, creating tissue necrosis. Pus consisting of necrotic tissue, dead bacteria within white blood cells (WBC) accumulates within the medullary cavity and increases the intramedullary pressure, generating drop in blood supply^6^. After saucerization and decortication surgical treatment, some inflammatory exudate with pathogenic bacteria is assumed to be retained after surgical closure, some of this retaining microorganisms and exudate are eventually phagocytized by the immune system. However, part of them might retained inflammatory exudate might remain in the wound area, causing wound healing delay and recurrence^8^. Therefore, decompression can be applied to the treatment of jaw OM, for reducing the intraluminal pressure, removing retained pathogenic bacteria, reduce swelling, pain, trismus and aid in the bone healing and bone regeneration process.

While the surgical drain eliminate pooled blood, pus, serum and edema reduction, exudate management, they promote drainage in the lymphatic vessels and dead space reduction of the surgical wound by drawing the separated surfaces together^10^. Following that, the drain reduces the edema and trismus. Decompression interventions are classified as open and closed with the closed one subdivided as passive or active. Active negative pressure drainage, also known as vacuum-assisted wound closure or negative-pressure wound therapy is a popular method for wound care including limb wounds, soft-tissue defects, chronic OM, osteofascial compartment syndrome, amputation, and replantation. Past research results have shown the effects of using negative pressure wound therapy in the head and neck region that include decreased healing time, less pain, and full drainage effects^11^. Despite the increasing number of studies of decompression, there are no reported studies of decompressive effects using drains in the management of jaw OM.

The purpose of this study was to investigate the effectiveness of decompression using a drain compared to management without drainage in a rat model of *S. aureus*-induced OM using micro-computed tomography (micro-CT) and histopathological analysis. The null hypothesis of this study is that decompression does not have therapeutic effects on infectious jaw OM and does not facilitate bone healing.

## Materials and methods

### Establishment of an *S. aureus-*infected jaw osteomyelitis rat model

Fourteen 8-week-old SPF Sprague–Dawley rats (OrientBio, Seongnam, Korea) weighing 230.13 (± 13.87) g on average were used in our study. The experimental protocols were approved by the Seoul National University (SNU) Institutional Animal Care and Use Committee (SNU-121123-12-11) and Institutional Biosafety Committee of SNU (SNUIBC-R121226-1-6). The experiment was in accordance with the “Recommendations for handling of Laboratory Animals for Biomedical Research” and complied with the Committee on Safety and ethical Handling Regulations for Laboratory Experiments at SNU. Animal studies were conducted following the ARRIVE guidelines for animal research^12^. The animal experiment was conducted at the Institute for Experimental Animals, College of Medicine, SNU, in a laboratory infection room classified as for high risk infection studies or infectious studies that use experimental animals (Animal Biosafety Level 2: ABL 2). All animals were maintained in an individually ventilated 12-h light/dark cycle cage system with the temperature ranging from 20 to 26 °C (23 ± 3 °C), and were provided rodent food and water ad libitum.

The bacterial strain used in our study was *S. aureus*, the most common causative pathogen for jaw OM^13^. We used a 2  to 8 °C freeze-dried *S. aureus* subsp. *Aureus* (ATCC 29213; American Type Culture Collection, Manassas, VA, US) and a Wichita designated clinical isolate that was provided by the Korean Culture Center of Microorganisms (KCCM, Seoul, Korea)^14^.

The suspended sample containing the *S. aureus* strain was then inoculated and spread with the spread method into a tryptic soy agar (TSA; Becton, Dickinson and Company, Franklin Lakes, NJ, US) plate medium using a sterilized inoculation loop and cultured in an incubator for 24 h at 37 °C^15^. After incubation, a visible colony of *S. aureus* formed. To determine bacterial density, we used the direct method of plate count technique (PCT) and the indirect method of turbidometry^16^. The number of bacterial inoculation was determined by PCT, in which the number of colonies formed on the plate medium is proportional to the live bacteria contained in the sample, and the dilution ratio and the number of colonies are calculated by stepwise dilutions.

**Figure 1:**
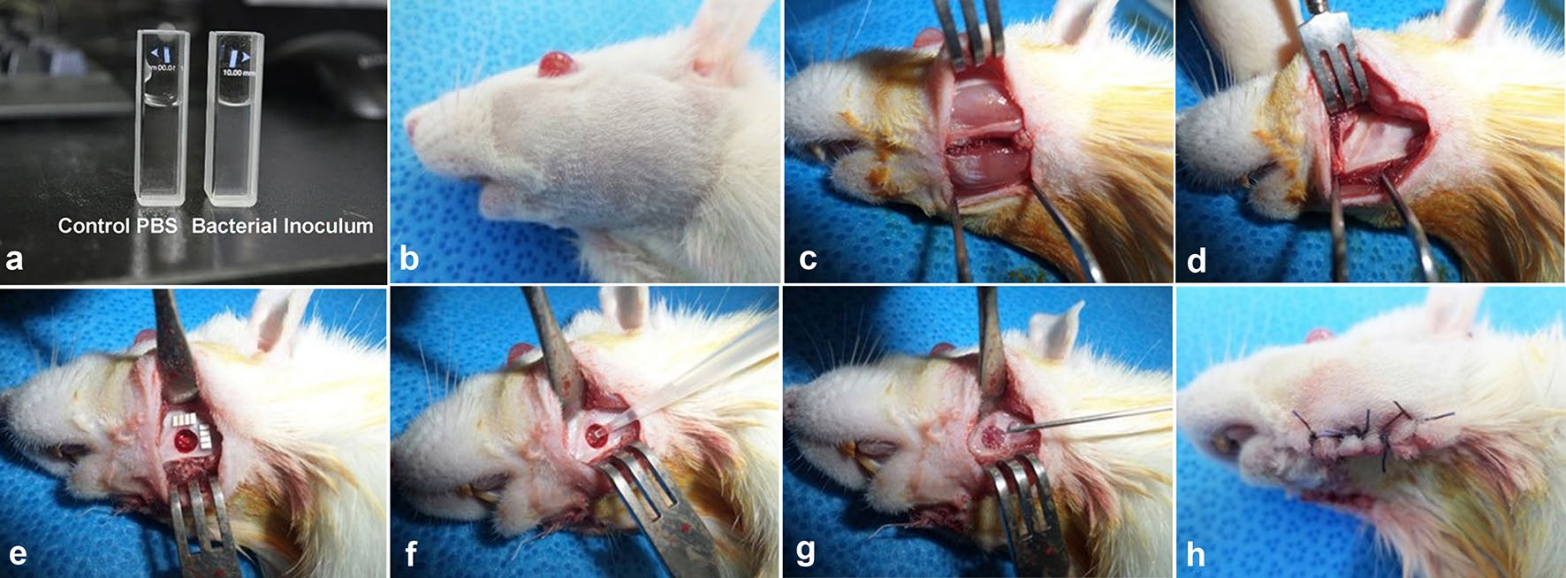
Inoculation process with *S. aureus* for inducing bacterial jaw OM. A UV/VIS spectrophotometer (LAMBDA 850 + UV/Vis Spectrophotometer; PerkinElmer, Waltham, MA, US) adjusted to an optical density 0.8 at 600 nm, that was used to measure the bacterial density for infection (**a**). The preparations for the surgical procedure including shaving, disinfection, and draping, which were all performed according to standard protocols (**b**). An approximately 12 mm full-thickness longitudinal extra-oral incision made parallel to the inferior border of the right and left side of rat mandibles. Adequate subcutaneous (**c**), deep fascial and periosteal dissections were performed followed by retraction with forceps (**d**). Using a low-speed hand piece with 1.2 mm diameter round bur, a bilateral circular 4 mm defect was created in the rat mandible (**e**) with copious irrigation. All animals received 20 μl of 10^7^ CFU/ml *S. aureus* injection (**f**) into the defect and were covered with fibrin glue (Greenplast Q; Green Cross, Yongin, Korea) (**g**). The surgical wound carefully sutured at the subcutaneous layer with resorbable 4-0 Vicryl (Polyglactin 910; Johnson & Johnson, Somerville, NJ, US) sutures and the skin closure was performed using silk sutures (BLACK SILK 4-0; AILEE, Busan, Korea) (**h**).

In the turbidity measurement, as the concentration of bacteria increases, the turbidity (absorbance) increases proportionally, therefore in order to measure turbidity as the actual number of bacteria, a correlation must be obtained. This can be obtained by measuring the number of bacteria with the direct plate count technique in parallel. The bacterial colony was harvested and was washed two times with phosphate-buffered saline (1 × PBS) by vortexing and by centrifuge. The suspended *S. aureus* solution was transferred to a new glass cuvette containing 1 × PBS and was adjusted to an optical density (OD) of 0.8 using a UV/VIS spectrophotometer (LAMBDA 850 + UV/Vis Spectrophotometer; PerkinElmer, Waltham, MA, US) at 600 nm with a clear PBS solution as a control (Fig. 1a). For the study, the TSA culture was diluted by 4 different OD values in four steps: (OD = 0.2) 1.1 × 10^6^; (OD = 0.4) 2.0 × 10^6^; (OD = 0.6) 4.5 × 10^6^; (OD = 0.8) 1.1 × 10^7^. The bacterial inoculation was then determined to be (OD = 0.8) 1 × 10^7^ CFU/ml at 600 nm, as the optimal bacterial amount required to induce jaw OM.

The infection with *S. aureus* was performed using a local inoculation route by injecting the bacterial suspension through the created defect^16^. The inoculation procedure was performed under general anesthesia using 90 mg/kg ketamine (50 mg/ml) (Ketamine; Yuhan, Seoul, Korea) + 10 mg/kg xylazine (23.32 mg/ml) (Rompun; Bayer Korea, Ansan, Korea) that was administered intraperitoneally^17^. The preparations for the surgical procedure including the skin preparation, disinfection, and draping were all performed according to standard protocols (Fig. 1b). An approximately 12 mm full-thickness longitudinal extra-oral incision was made parallel to the inferior border of the right and left side of rat mandibles. Adequate subcutaneous (Fig. 1c), deep fascial and periosteal dissections were performed followed by retraction with forceps (Fig. 1d). Using a low-speed hand piece with 1.2 mm diameter round bur, a bilateral circular 4 mm defect was created in the rat mandible (Fig. 1e) with copious irrigation. The defect was made from the buccal side, inferior to the incisor tooth root, posterior to the second molar, and at the attachment site of the superficial masseter muscle. Considering the anatomy of the rat and the objective of the study, a circular 4 mm defect is a generally accepted mandibular bone defect^18^.

All animals received 20 μl of 10^7^ CFU/ml *S. aureus* injection (Fig. 1f) into the defect and were covered with fibrin glue (Greenplast Q; Green Cross, Yongin, Korea) to prevent bacterial leakage (Fig. 1g)^19^. The surgical wound was then carefully sutured at the subcutaneous layer with resorbable 4-0 Vicryl sutures (Polyglactin 910; Johnson & Johnson, Somerville, NJ, US) and the skin closure was performed using 2-0 silk sutures (BLACK SILK 4–0; AILEE, Busan, Korea) (Fig. 1h).

### Grouping and experimental design

The animals were randomly divided into control (non-decompression groups, C1 and C2) and experimental groups (decompression groups, E1 and E2) (Table 1). The C1 group (n = 3) served as the control group, who only received wound closure. The C2 control group (n = 4) received conventional surgical curettage for jaw OM.

**Table 1:** Animal grouping.

Group	Treatment	Sacrifice period
C1	Non-decompression group, suturing after incision	4 weeks
C2	Non-decompression group, debridement of necrotic tissue and curettage	4 weeks
E1	Debridement of necrotic tissue and draining tube insertion after curettage	4 weeks
E2	Debridement of necrotic tissue and curettage followed by draining tube insertion and normal saline irrigation at 1-week intervals	4 weeks

The animals were divided into control groups (C1 and C2) and experimental groups (E1 and E2).

*CFU* Colony Forming Unit.

The experimental groups were further classified into two subgroups, E1 group (n = 3), which received removal of pus and necrotic bone tissue and curettage, followed by introducing the tube drain and E2 group (n = 4), which received removal of pus and necrotic bone tissue and curettage, drain insertion and irrigation with normal saline every week.

Blood samples were collected from the tail vein pre-infection, 1-week post-infection, 2-weeks post-infection/the start of the treatment, 1-week after treatment, and 4-weeks after the treatment, and rat weights were checked.

Surgical treatment was performed under same general anesthesia protocol used in the inoculation procedure that mentioned in the previous section of materials and methods^17^. Two mm inner diameter and three mm outer diameter silicone tubes that were approximately 2 cm in length (Translucent PFA Tubing; DAIHAN Scientific, Wonju, Korea) were used as a drain. The length of the tube was adjusted to each animal according to the post-curettage conditions and these were sutured in place using 4-0 silk sutures. To keep the tubes intact and in place, we used a plastic collar to prevent scratching and accidental displacement of the draining tubes. The animals that died during the experimental trial were recorded for weight loss and clinical symptoms of OM of the jaw.

Upon the completion of the six-week experimental trial, the animals were euthanized by CO_2_ inhalation. The mandibles of the rats were immediately harvested and carefully isolated.

### The analysis of bone healing with micro-CT

The rat mandible specimens were subjected to high-resolution micro-CT scanning Skyscan 1172 (SKYSCAN 1172; Bruker, Kontich, Belgium). The scanning parameters of the source were adjusted to an Al filter of 0.5 mm, source voltage of 70 kV, source current of 141 μA, and 360° rotations at 0.4° rotation steps. This resulted in images that were 496 pixels in width and 900 pixels in height.

Following the scanning procedure, the raw data sets were reconstructed using NRecon 1.6.9.8 (NRecon 1.6.9.8; Bruker, Kontich, Belgium) software. The smoothing was adjusted to 6, ring artifact correction to 7, with a beam hardening correction to 10%.

**Figure 2:**
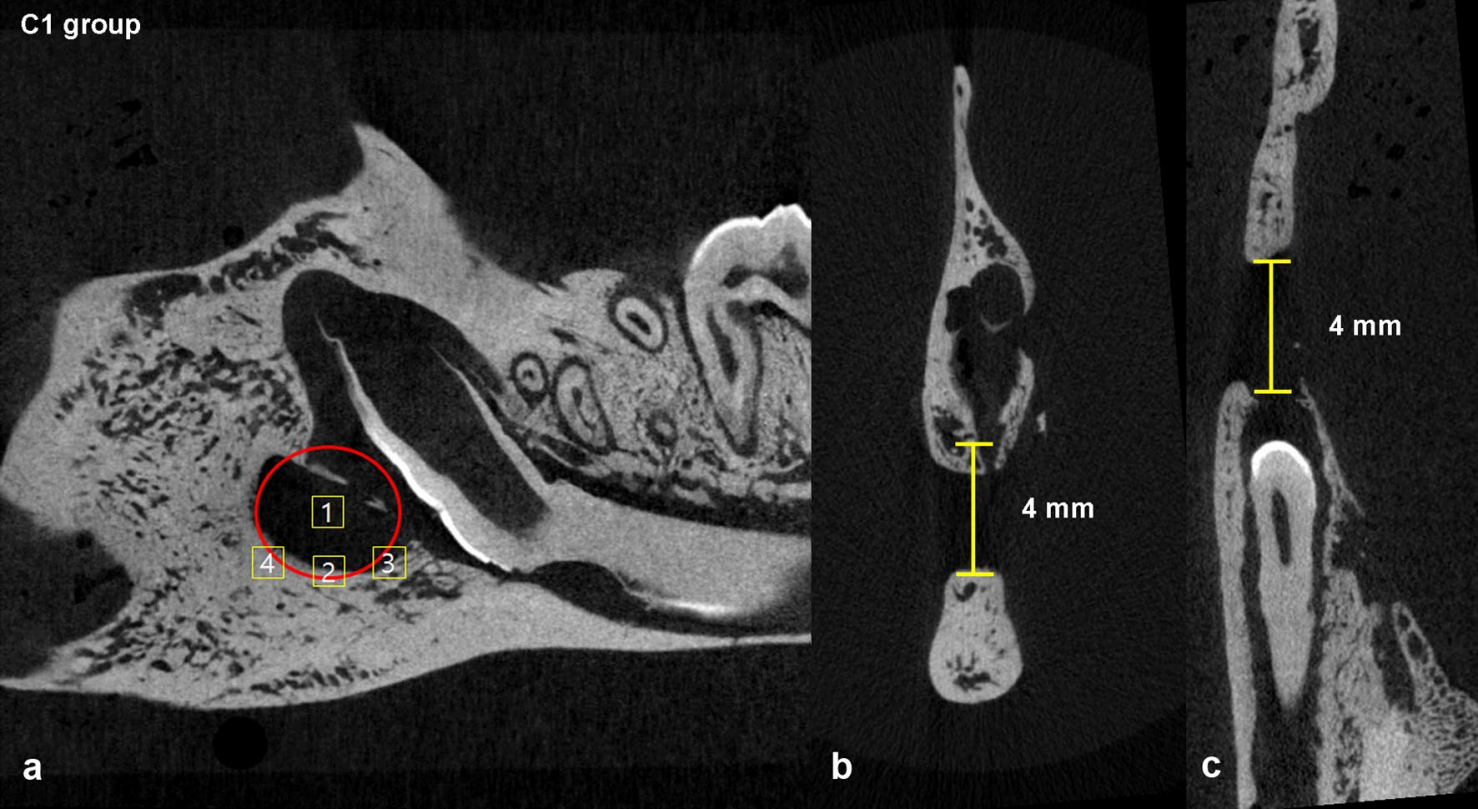
ROI designation method at the round 4 mm defect area. A circular area with a 4 mm wide diameter was first set in the sagittal plane that depicted the defect and four 1 × 1 mm square ROIs were set at the center of the defect and the inferior margins of the circular area (**a**). Axial plane showing bone defects and bone destruction at the buccal surface of the rat mandible (**b**). Coronal view showing the bone defect marked with yellow color (**c**).

Each dataset was opened and further adjusted using DataViewer (DataViewer; Bruker, Kontich, Belgium) software. The region of interest (ROI) were determined in the sagittal plane and the image analysis were performed using CTAn software (CTAn version 1.18.4.0, Bruker, Kontich, Belgium). Incisor roots were excluded from the analysis and only bone tissue was included for bone analysis. Equivalent thresholds were adjusted in all images. To determine the ROI, a 4 mm wide circular area was set up in the sagittal plane where the initial 4 mm bone defect area could be seen. For optimal comparison between the samples, an identical number of slices were selected. Four square shaped ROI’s were defined as 1.0 mm in width and 1.0 mm in height that were adjusted for analysis at the center and the inferior borders of the circular area as seen in Fig. 2. The same procedure was performed on the contralateral rat mandible. Within the ROI, bone mineral density (BMD, g/cm^3^), bone volume (BV, mm^3^), and bone volume/volume of interest (BV/VOI, %), bone surface (BS, mm^2^), bone surface/volume ratio (BS/BV, 1/mm), trabecular thickness (TB.Th., mm), trabecular number (Tb.N, 1/mm), and trabecular separation (Tb.Sp., mm) were measured and compared. The datasets were reconstructed into three dimensional (3D) images using CTvox volume rendering software (CTvox; Bruker, Kontich, Belgium).

### The histological and immunohistological analysis of OM healing

The samples from each group were trimmed and decalcified with 0.5 M ethylene diamine tetra-acetic acid (pH 8.0) (0.5 M EDTA, pH 8.0; BIOSESANG, Sungnam, Korea) solution for ten days, dehydrated with 70% ethanol, and embedded into paraffin. The 4 μm thick slides were then washed with xylene for approximately 10 min and were stained with hematoxylin and eosin (H&E) and Masson’s trichrome (MT). The histological slides were then scanned with a 3D scanner (PANNORAMIC 250 Flash III; 3DHISTECH, Budapest, Hungary) and examined using slide-viewing software (CaseViewer version 2.0; 3DHISTECH, Budapest, Hungary).

**Figure 3:**
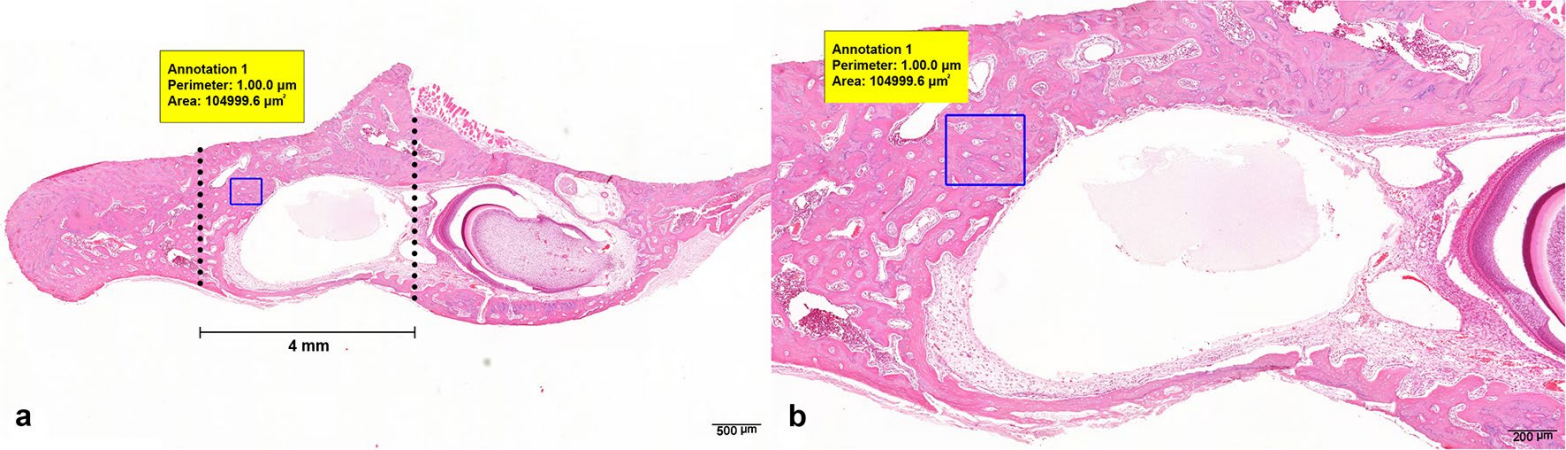
Osteocyte and Haversian canal count method, 2 × (**a**). At the center of the defect area with bone regeneration, a 500 × 500 μm fixed rectangle was selected as the area of interest for counting, 5 × (**b**).

For quantitative analysis, the number of osteocytes and Haversian canals within the regenerated bone tissues of the defect area were counted. The 4 mm circular defect area was determined from the micro-CT 3D images. An area of interest using a fixed rectangular form of 350 × 300 μm within the initial defect area was established by the histological slide-viewing software (CaseViewer version 2.0; 3DHISTECH, Budapest, Hungary) in all of the specimens at a magnification of 20 × (Fig. 3).

Paraffin-embedded samples were cut into a thickness of 4 μm and were mounted on glass slides. The examination was performed by using a light microscope (OLYMPUS BX41; OLYMPUS, Tokyo, Japan). For immunohistochemistry (IHC) staining we used vascular endothelial growth factor A (VEGF-A) (ab46154; Abcam, Cambridge, MA, US) at 1:100 dilution, Transforming growth factor β1 (TGF-β1) (sc-130348; Santa Cruz Biotechnology, Dallas, TX, US) at 1:100 dilution, Osteopontin (OPN) (sc-73631; Santa Cruz Biotechnology, Dallas, TX, US) at 1:100 dilution, Alkaline Phosphatase (ALP) (sc-271431; Santa Cruz Biotechnology, Dallas, TX, US) at 1:100 dilution, TNF-α (300-01A; PeproTech, Cranbury, NJ, US) at 1:100 dilution, and IL-6 (sc-28343; Santa Cruz Biotechnology, Dallas, TX, US) at 1:100 dilution antibodies. The staining was scored as follows: “1”: none, “2”: 1–25%, “3”: 26–50%, “4”: 51–75%, and “5”: 76–100% cells stained^20^. The intensity of the antibody staining was assessed using a previously described method^21^.

### Statistical analysis

Means and standard deviations for bone healing parameters were obtained. The data normal distribution was tested by Shapiro–Wilk test and showed homogeneity. The differences between groups were tested by ANOVA followed by Tukey–Kramer multiple comparison tests. Statistical analyses were done using SPSS for Windows, version 25.0 (IBM SPSS Statistics; IBM, Armonk, NY, US). *P* < 0.05 were considered statistically significant.

### Ethical approval

The experimental protocols were approved by the Seoul National University (SNU) Institutional Animal Care and Use Committee (SNU-121123-12-11) and Institutional Biosafety Committee of SNU (SNUIBC-R121226-1-6). The experiment was in accordance with the “Recommendations for handling of Laboratory Animals for Biomedical Research” and complied with the Committee on Safety and ethical Handling Regulations for Laboratory Experiments at SNU. Animal studies were conducted following the ARRIVE guidelines and are in accordance with the 1964 Helsinki declaration and its later amendments or comparable ethical standards.

## Results

### Establishment of an *S. aureus-*infected jaw osteomyelitis rat model

The pathogen dose of 20 μl of 10^7^ CFU/ml was effective in creating jaw OM in the rat model and a repeatable animal model was established. After two weeks of infection, all groups showed visible clinical manifestation of infectious jaw OM including: skin redness, swelling, purulent discharge and alopecia. Six animals died during the experimental trial due to OM complication including, one animal from the C1 group, three animals from the C2 group, one animal from the E1, and one animal from E2. Three animals that died from the C2 group, which received conventional surgical curettage for jaw OM without decompression, could be explained by the development of bacterial jaw OM and the surgical intervention that can cause serious adverse burden on the body. Consequently, after six weeks, the specimens of the remaining animals (n = 8) were collected and were further analyzed. All of the animals were in good health before the infection. The clinical findings showed common characteristics of the jaw osteomyelitis. The establishment of *S. aureus-*infected jaw osteomyelitis rat model were confirmed by the following parameters: clinical findings, blood test, micro-CT bone architecture findings, and histological analysis.

### Clinical evaluation with blood test

After the infection with *S. aureus* all animals from the control and the experimental groups showed weight loss. Significant weight loss in all groups was observed with an average of −31.57 (± 21.98) g at one week after the infection that was recovered after two weeks (*p* = 0.000). There was no statistical significance in weight loss between the groups at 1 week after infection (Table 2).

**Table 2:** Weight changes after infection with S. *aureus* and treatment in the control and experimental groups.

Group	Before infection (g)	1 week after infection (g)	2 weeks after infection (g)	1 week after treatment (g)	4 week after treatment (g)
C1	223.63 (± 4.12)	190.40 (± 11.47)	186.63 (± 42.78)	211.90 (± 40.58)	245.75 (± 4.03)
C2	223.35 (± 8.00)	177.30 (± 20.30)	210.35 (± 25.81)	198.40	259.90
E1	246.80 (± 19.47)	211.0 (± 5.63)	218.17 (± 33.43)	243.30 (± 7.07)	275.30 (± 19.37)
E2	229.27 (± 11.10)	216.60 (± 17.41)	236.42 (± 20.96)	260.46 (± 7.59)	294.60 (± 11.51)

The weight changes are depicted as mean ± standard deviation (SD).

The neutrophil count was significantly increased after the infection in all groups. There were no statistically significant differences in neutrophil count between the groups at one week and four weeks after treatment (Table 3). The WBC count was significantly increased in all groups after infection and was recovered to the normal range at four weeks after treatment (*p* = 0.04). No significant difference was observed between the groups at one week after treatment and four weeks after treatment (Table 4). Serum levels of alkaline phosphatase (ALP) were also measured and analyzed. At one week after infection, ALP levels were significantly increased in all groups (*p* = 0.001) and a significant reduction was observed at one week and two weeks after treatment (*p* = 0.004, *p* = 0.000, respectively). No significant differences were found between groups (Table 5).

**Table 3:** The change of the Neutrophil percentage.

Group	Before infection	1 week after infection	2 weeks after infection	1 week after treatment	4 week after treatment
C1	12.20 (± 3.11)	33.45 (± 4.45)	29.26 (± 7.82)	32.50 (± 3.53)	13.00 (± 4.94)
C2	28.52 (± 15.13)	35.87 (± 19.79)	37.00 (± 2.68)	29.10	7.90
E1	22.75 (± 15.12)	30.36 (± 5.71)	20.60 (± 4.10)	34.40 (± 9.30)	12.77 (± 4.08)
E2	16.00 (± 6.29)	31.95 (± 2.49)	34.12 (± 7.31)	28.96 (± 9.00)	13.16 (± 2.26)

The changes are depicted as mean ± standard deviation (SD).

**Table 4:** Changes of WBC count.

Group	Before infection	1 week after infection	2 weeks after infection	1 week after treatment	4 week after treatment
C1	8.70 (± 2.74)	9.15 (± 3.41)	17.10 (± 4.28)	16.47 (± 3.73)	8.11 (± 1.27)
C2	7.51 (± 0.57)	12.52 (± 3.05)	17.43 (± 9.01)	13.37	9.65
E1	12.52 (± 0.67)	13.31 (± 3.88)	14.63 (± 3.93)	16.50 (± 7.02)	10.46 (± 1.91)
E2	11.19 (± 2.39)	17.01 (± 7.61)	20.09 (± 3.42)	18.38 (± 4.50)	11.77 (± 0.62)

The changes are depicted as mean ± standard deviation (SD).

**Table 5:** Results of ALP changes.

Group	Before infection	1 week after infection	2 weeks after infection	1 week after treatment	4 week after treatment
C1	535.33 (± 77.50)	957.66 (± 185.30)	957.31 (± 957.31)	526.00 (± 42.42)	389.50 (± 6.36)
C2	702.75 (± 107.77)	1512.25 (± 267.35)	1110.5 (± 272.09)	526.00	488.00
E1	817.00 (± 179.67)	1374.00 (± 231.63)	957.31 (± 213.98)	588.00 (± 132.82)	407.33 (± 65.63)
E2	739.66 (± 179.66)	984.25 (± 302.69)	860.00 (± 65.79)	718.00 (± 117.20)	390.33 (± 101.20)

The changes are depicted as mean ± standard deviation (SD).

### Micro-CT results of bone healing

From the 3D images, more bone healing was observed in the E1 and E2 groups, where the initial bone defect was replaced by new bone tissue. The E2 group had the most compact bone formation compared to the other groups. The C1 group, which received no treatment, showed bone destruction that continuously spread from the initial defect affecting a wider area. The common characteristics of osteomyelitis of the jaw such as the bone necrosis and sequestrum formation, a segment of necrotic bone that is separated from the viable bone by a granulation tissue and bone resorption was observed from the control groups.

**Figure 4:**
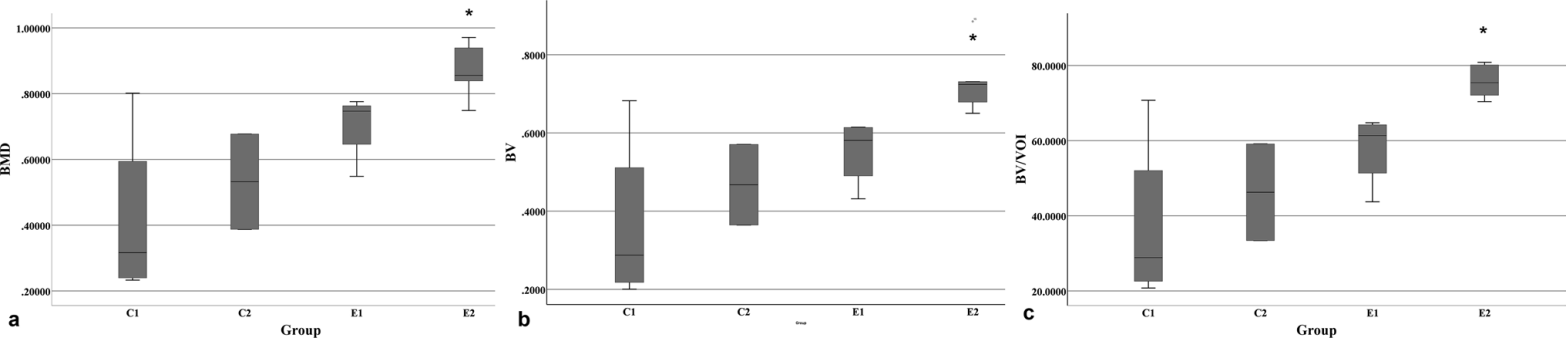
BMD differences between the groups. The E2 group was significantly higher than the C1 group (*p = 0.004) (**a**). The BV and BV/VOI was significantly higher in the E2 group than in the control groups (*p = 0.005, *p* = 0.003, respectively), while the E1 group showed no significant differences with other groups (**b**, **c**).

The BMD results were significantly different between the groups. The BMD in the C1 group was significantly lower compared to that of the E2 group, with a mean difference of -0.45 g/cm^3^ (*p* = 0.004) (Table 6) (Fig. 4a).

**Table 6:** Micro-CT morphometric parameters of the bone regeneration area in 3D analysis.

Group	BMD (g/cm^3^)	BV (mm^3^)	BV/VOI (%)	BS (mm^2^)	BS/VOI (1/mm)	Tb.N (1/mm)	Tb.Sp (mm)
C1	0.42 (± 0.27)	0.36 (± 0.22)	37.31 (± 24.59)	5.52 (± 2.42)	5.83 (± 2.81)	1.51 (± 0.74)	0.46 (± 0.19)
C2	0.53 (± 0.20)	0.47 (± 0.14)	46.26 (± 18.19)	7.91 (± 1.79)	8.04 (± 2.09)	2.24 (± 0.53)	0.34 (± 0.10)
E1	0.70 (± 0.10)	0.55 (± 0.08)	57.79 (± 19.53)	9.80 (± 0.32)**	10.70 (± 0.80)**	2.78 (± 0.52)**	0.17 (± 0.01)**
E2	0.87 (± 0.08)*	0.73 (± 0.08)*	75.70 (± 14.32)*	11.07 (± 1.75)*	11.44 (± 1.43)*	3.28 (± 0.66)*	0.12 (± 0.01)*
P value	0.007	0.008	0.006	0.002	0.001	0.008	0.001

The data is depicted as mean ± standard deviation (SD)

*BMD* bone mineral density, *BV* bone volume, *BV/VOI* bone volume/volume of interest, *BS* bone surface, *BS/VOI* bone surface/volume of interest ratio, *Tb.N* trabecular number, *Tb.Sp* trabecular spaces.

*p < 0.05, E2 group versus the C1 group.

**p < 0.05, E1 group versus the C1 group.

**Figure 5:**
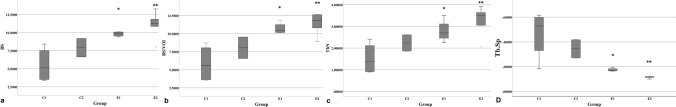
Comparison of micro-CT parameters between the groups. BS (*p = 0.013, **p = 0.003, respectively) (**a**), bone surface and BS/VOI (**b**) (*p = 0.012, **p = 0.002, respectively), Tb.N (**c**) (*p = 0.031, **p = 0.004, respectively), Tb.Sp (**d**) (*p = 0.026, **p = 0.002, respectively).

The BV was highest among E1 and E2 groups. The E2 group showed significantly higher results compared to that of the C1 group (*p* = 0.005) with a mean value of 0.73 (± 0.08) mm^3^ (*p* < 0.05) (Fig. 4b). The BV/VOI parameter was significantly higher in the E2 group, with an average value of 75.70 (± 14.32) % compared to that of the C1 group (*p* = 0.003) (Fig. 4c). The BS, BS/VOI, Tb.N, Tb.Sp parameters were significantly different between the control and experimental groups (Table 6). Bone healing parameters such as BS, BS/VOI, Tb.N showed significant difference between the control and experimental groups (*p* = 0.002, *p* = 0.001, 0.008, respectively) (Fig. 5a–c). However, the Tb.Sp in the C1 group was significantly higher than that in the experimental groups (Fig. 5d). The Tb.Th was not significantly different between the groups.

**Figure 6:**
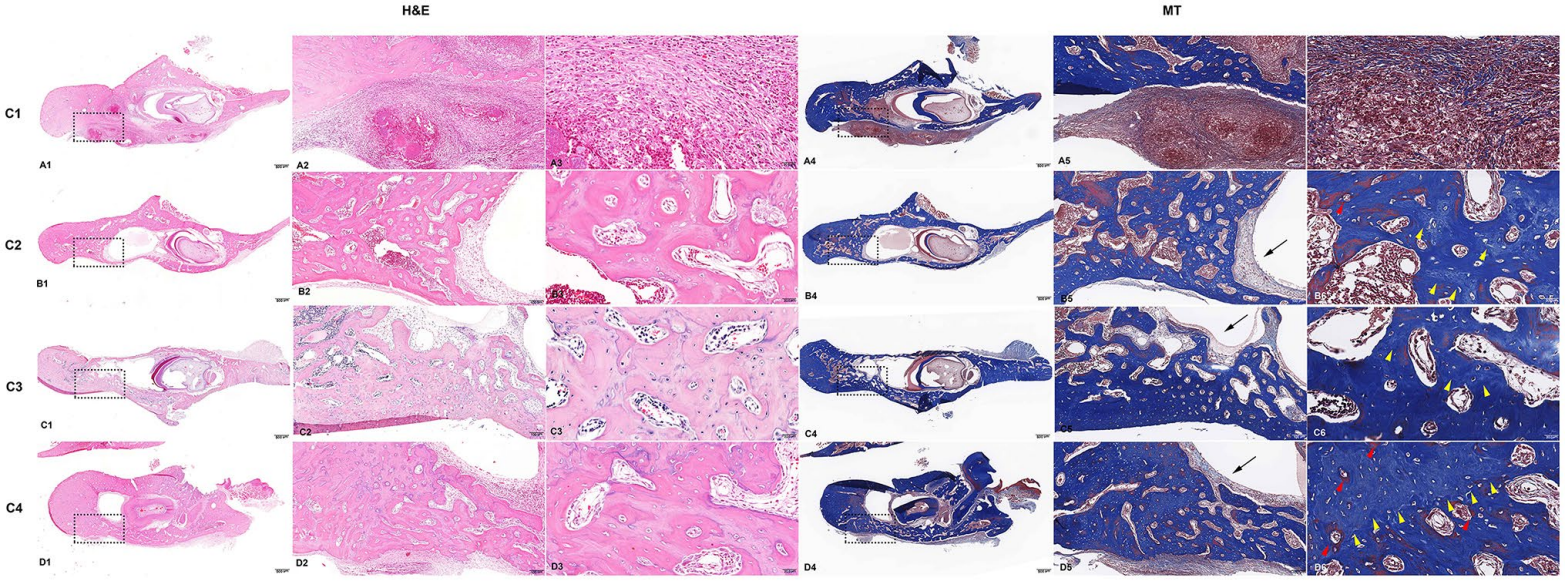
Representative histological images of the specimens following 4 weeks of treatment stained with H&E and MT. In the C1 group, intense inflammatory infiltration is observed, 5.0 ×  (**A1**). Magnification of the rectangles, 10 × , 40 × (**A2**–**A3**). MT stain for bone regeneration in the defect area (**A4**). Magnification of the rectangles, 10 × , 40 × (**A5**–**A6**). The C2 group showed bone healing with osteoblastic cell lining in the parenchymal tissue found at the center of the defect area with evidence of inflammatory infiltrates (**B1**). Magnification of the rectangles, 10 × , 4 0 × (**B2**–**B3**). MT stain for bone regeneration in the C2 group at 2 × (**B4**). Magnification of the rectangles, 10 × , 40 × (**B5**–**B6**). The E1 group showed loose marrow fibrosis and scattered lymphocytic inflammatory infiltrates (**C1**). High power view of the rectangles, 10 × , 40 × (**C2**, **C3**). The MT stain of E1 group showed new bone formation stained with bright blue color and new blood vessels were observed (**C4**). Magnification of the rectangles, 10 × , 40 × (**C5**, **C6**). The E2 group showed increased osteophytic bone formation (**D1**). High power view of the rectangles at 10 × , 40 × (**D2**, **D3**). The E2 group showed active bone remodeling with the thickest compact new bone formation in the defect area compared to that of other groups (**D4**). An increased number of Haversian canals with osteoblast rimming and new blood vessel formation stained with thick red color by the MT stain were observed at 10 × , 40 × magnification (**D5**, **D6**). Black arrow, parenchymal tissue, Yellow arrowhead, new bone formation, Red arrow, blood vessel formation.

### Histological and immunohistochemical results of OM healing

The morphological changes in bone healing were macroscopically observed in the H&E and MT stained slides at 4-weeks in the defect area.

The C1 group showed high grade inflammatory infiltration consisting of neutrophils, eosinophils, and macrophages around the bacterial colonies. Signs of infective osteomyelitis including bone necrosis, bone resorption and destruction, with no bone healing were observed. In MT staining, the C1 group were stained with thick blue color, indicating old bone, while no new bone formation stained with bright blue color were observed (Fig. 6A1–A6). Histological findings for the C2 group showed bone healing with osteoblastic cell lining in the parenchymal tissue found at the center of the defect area with evidence of inflammatory infiltrates (Fig. 6B1–B6).

The histology features of the E1 group included scattered lymphocytic inflammatory infiltrates and loose marrow fibrosis. In the MT stain, new bone formation stained with bright blue color and new blood vessels were observed (Fig. 6C1–C6). The E2 group showed active bone remodeling with the thickest and most compact new bone formation being in the defect area compared to that of other groups. Increased osteophytic bone formation was observed. Furthermore, an increased number of Haversian canals with osteoblast rimming and new blood vessel formation stained with thick red color by the MT stain were seen (Fig. 6D1–D6).

We counted the number of osteocytes and Haversian canals in the ROI for quantitative analysis. The results showed that the E2 groups had a statistically significant greater osteocyte count compared to the control groups (*p* = 0.002) (Table 7).

**Table 7:** Osteocyte and Haversian canal count in the ROI.

Group	Osteocyte	Haversian Canal
C1	60.5 (± 10.1)	5.75 (± 3.30)
C2	53.5 (± 9.20)	8.00 (± 1.40)
E1	77.25 (± 8.40)	8.50 (± 2.90)
E2	91.00 (± 9.90)*	9.75 (± 2.10)

The data is depicted as mean ± standard deviation (SD). *The osteocytes found in the E2 group were significantly higher than in the C1 and C2 groups, *p* = 0.002).

In order to confirm the inflammatory, angiogenic, and osteogenic properties in the control and experimental groups, IHC staining was performed. The expression of inflammation-related antibody IL-6 in the E2 group was weak (score “2”: 1–25% of cells positive), compared with that of the C1 group (“5”: 76–100% cells stained). TGF-β1 expression was markedly high in the E1 group, while the C1 group showed no expression (“1”: none). The TNF-α antibody stained strongly in the C1 and C2 groups compared to that in the other groups (“5”: 76–100% cells stained).

**Figure 7:**
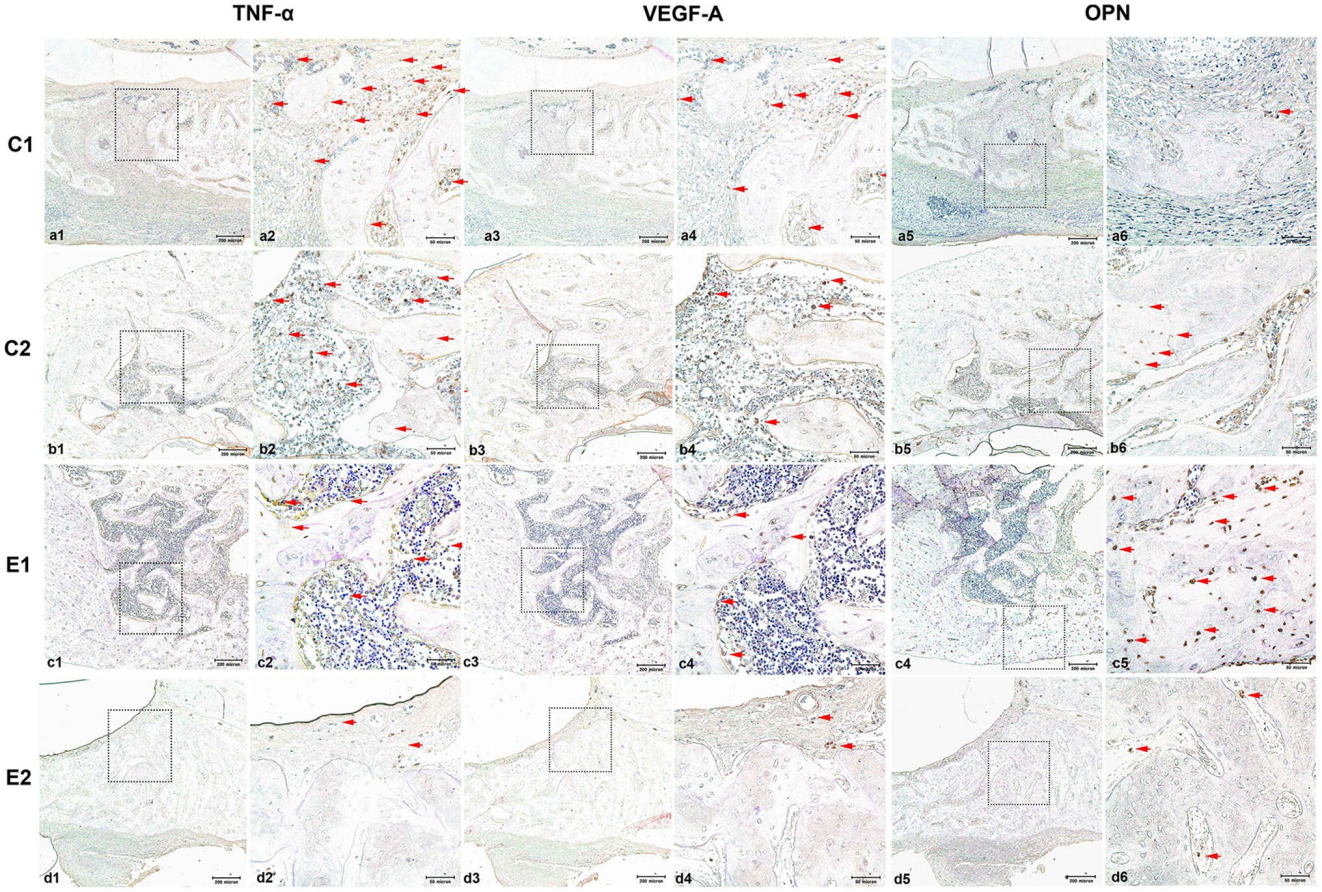
TNF-α, VEGF-A, and OPN antibody staining in the C1 group (**a1** to **a6**), C2 group (**b1** to** b6**), E1 group (**c1** to **c6**), and E2 group (**d1** to **d6**). TNF-α staining with original magnification, 10 × (**a1**), Magnified image from the selected region from A1, 40 × (**a2**). VEGF-A staining with original magnification, 10 × (**a3**), Magnified image from the selected region from **a3**, 40 × (**a4**). OPN staining with original magnification, 10 × (**a5**), Magnified image from the selected region from a5, 40 × (**a6**). TNF-α (**b1** to **b2**), VEGF-A (**b3** to **b4**), and OPN (**b5** to **b6**) antibody staining in the C2 group with original magnifications, 10 × , 40 × , respectively. TNF-α (**c1** to **c2**), VEGF-A (**c3** to **c4**), and OPN (**c5** to **c6**) antibody staining in the E1 group with original magnifications, 10 × , 40 × , respectively. TNF-α (**d1** to **d2**), VEGF-A (**d3** to **d4**), and OPN (**d5** to **d6**) antibody staining in the E2 group with original magnifications, 10 × , 40 × , respectively. The red arrowheads mark the stained antibodies in the defect area.

The expression of VEGF-A was the highest in C1 (“5”: 76–100%) compared to that of E2 (“2”: 1–25%). The osteogenesis markers, ALP and OPN, were also strongly expressed in the E1 group compared to that seen in other groups (Fig. 7).

## Discussion

The result of current study showed that decompression had significantly greater bone healing properties compared to the conventional surgical treatment alone in infectious jaw OM, based on the clinical, micro-CT, histological and immunohistochemical analysis. Thus, the null hypothesis was rejected. Decompression promotes wound healing through the increased upregulation of innate immunity-related proteins, osteogenic and angiogenic proteins after decompression usage in the postoperative jaw OM wound area^7^. To the best of our knowledge, the current study analyzes for the first time the decompression effects of using drain in vivo jaw OM model.

Decompression using drains is a well-established and a reliable conservative treatment method for cystic lesions in the jaw. Although this method is used often for treating cystic lesion and reported extensively for its therapeutic effects in the literature, there are no studies of decompression treatment used for jaw OM cases. After surgical treatments for jaw OM, such as saucerization or decortication, excess fluid build-up can exert high pressure within the bone marrow covered by the cortical bone. Surgeons usually implement surgical drains to remove any excess postoperative exudate to prevent edema formation. However, the same bone healing and bone regenerative effects of decompression can be applied to the treatment jaw OM. Therefore, this study will be discussing about therapeutic effects of decompression in the jaw OM, rather than decompression in cystic lesion.

The effectiveness of decompression in the treatment of jaw OM can be explained by fluid removal and alteration of the wound environment to be conducive to healing. This excess fluid built-up with high pressure can be regarded as one of the major factors that compromise healing, partly owing to the compressive pressure that it exerts on local cells and surrounding tissue^22^. If the fluid pressure is elevated in the bone marrow, the proliferative response diminishes due to dampened intrinsic tension build-up. Applying decompression and drainage to this area permits fluid removal from the extracellular space. The removal of postoperative fluid allows decompression of the microvasculature that permits tissue perfusion by reducing pressure and enhancing blood circulation to the area. It will also remove the toxins, inflammatory exudate, and pathogenic bacteria from the operative site, which is considered to be an important element in the wound healing process.

The significance of this study is that it demonstrates the effectiveness of decompression using a drain in jaw OM, which had significant bone healing effects according to micro-CT, histology, and IHC analyses. For developing new therapeutic methods for OM of the jaw, it is important to establish standard operative protocols for animal modeling^23^. To our knowledge, there are no scientific data in the literature on decompression effects using drain in jaw OM animal model. Also, the current *S. aureus*-infected jaw osteomyelitis rat model has not been previously described in the literature before.

**Figure 8:**
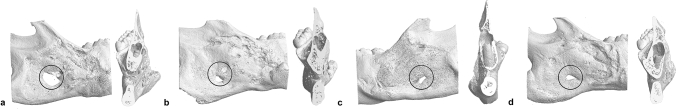
3D reconstructed images of the C1 (**a**), C2 (**b**), E1 (**c**), and E2 (**d**) groups. Images show the enhanced bone healing in the round defect in the E1 and E2 groups.

We report our methodology according to the guidelines for assessment of bone microstructure in rodents using micro-CT^24^ and results by following morphometric indices that can determine new bone formation^25^. The most informative parameters which show the course of bone healing are BV, BV/VOI, BS/VOI, and BMD^24,26^. More pronounced increase of BV, BV/VOI, BS/VOI, and BMD results were achieved in the experimental E1 and E2 groups compared to that of the control groups. The 3D evaluation showed more rapid bone healing was detected in the E1 and E2 groups compared to that of the C1 and C2 groups (Fig. 8). This can be explained by the quantity and the quality of newly formed bone in the defect area of jaw OM rat model. These results were supported by the histology analysis, where the control groups had evident inflammatory infiltration with bacterial colonies, making it unfavorable for bone healing and bone regeneration. The IHC results also in accordance with the micro-CT findings, which show strong staining results osteogenesis-related proteins ALP and OPN. This favorable outcome suggests that decompression in combination with the surgical treatment can remove the post-operative exudate built-up, removing any inflammatory toxins from the wound and decrease the pressure from high pressure area, thereby supporting bone healing and bone regeneration.

In terms of bone healing and anti-inflammation, the experimental groups showed more effective results that included rapid bone healing, blood vessel formation, and reduced inflammation. The E2 group also had the highest osteocyte count. An increase in osteocytes plays a pivotal role in regulating bone turnover, which also enhances osteogenesis of stem cells, suggesting an important role in tissue regeneration^27^. The histological results suggested the most optimal bony healing was seen in the experimental groups, which is in accordance with the micro-CT analysis.

In our study we demonstrated that VEGF-A was activated and then reduced in the E2 group, leading to enhanced and accelerated bone healing compared to the E1 group, while bone healing was evident, but much slower. Angiogenesis and osteogenesis are two intimately connected processes that must be closely coupled to permit physiological bone function. In fact, alterations in vascular growth can alter the physiological bone healing process, which may lead to osteoporosis, osteonecrosis, and non-union fractures^28^. According to a previous clinical study, decompression had a direct influence on the microvascular circulation, and enhanced VEGF protein in the first day following surgical treatment, thereby activating osteogenesis-related proteins such as OPN and ALP was decreased on the second day^8^.

In the bone remodeling phase, TNF-α and other pro-inflammatory cytokines are thought to play an important role in bone healing^29^. IL-6 is a well-known cytokine that stimulate osteoclast differentiation and bone resorption indirectly depending on the context of release^30^. TNF-α is a widely known key player in the pathogenesis of osteomyelitis. In IHC staining, the pro-inflammatory antibodies IL-6 and TNF-α were strongly stained in the C1 group, indicating an inflammatory reaction to the bacteria, while the groups which received surgical treatment were weakly stained. From the Micro-CT and histology analysis, C1 and C2 groups exhibited the weakest bone healing with strong inflammatory infiltration, which could be explained by the high expression of IL-6 found in C1, and high expression of TNF-α in the C1 and C2 groups. The high expression of IL-6 and TNF-α found in these groups had active bone resorptive effects, which suppressed the osteogenesis.

The high expression of TGF-β1 found in the E1 group indicate active angiogenic function of decompression as well reduced activation of osteoclasts and bone resorption. Compared to E1 group, the E2 group showed well-formed cortical bone with less marrow bone. This could also explain the weak stain of angiogenesis and osteogenesis related antibodies, since adequate and compact bone healing was already established compared to that of other groups. TGF-β is an important cytokine that balances bone formation and bone resorption, mineral storage, hematopoietic cell generation and osteoimmunology^31,32^. Especially, high concentration of TGF-β1 reduces the activation of osteoclasts, while low concentrations promotes osteoclast maturation^33^. The TGF-β1 was expressed in high expression in the E1 group. Previous studies suggest that TGF-β1 prevents TNF-α-induced bone destruction by suppressing effecter T cell function^32^.

The results were in accordance with our hypothesis that decompression using a drain had significant therapeutic effects on bone regeneration for jaw OM, therefore the null hypothesis is not rejected. When decompression was applied to the curettage treatment, enhanced wound and bone healing were achieved. The decompression effects on the healing process were much enhanced with weekly normal saline irrigation. Although many surgeons use decompression technique for managing cystic lesions, their use of managing jaw OM is not known in the literature. The current study results strongly support the clinical relevance of decompression in combination with the conventional surgical treatment for jaw OM. From our ongoing clinical study, this treatment method has many clinical merits, such as reducing swelling, discomfort, easy to use, convenient and economical^34^. The results of this clinical study revealed that the group of patients treated with saucerization and drain insertion exhibited more enhanced bone density compared to the groups without drainage at the six-month and one-year follow-ups. In addition, the drain insertion for decompression show the effectiveness in both sclerosing and suppurative types OM in the jaw.

In the future, the current model can be enhanced by additional design of an intervention group with antibiotic treatment, for studying this combination of surgical decompressive, and medical treatment. In this way, the most effective treatment protocol for each type of OM can be determined. At present, the application of decompression could be a reliable choice for the surgeons and clinicians as a treatment in combination with surgical treatment to allow accelerated bone healing.

## Data Availability

The datasets generated during and/or analyzed by the authors during this study are available from the corresponding author on reasonable request.
